# Recent Advances in Biological Activity, New Formulations and Prodrugs of Ferulic Acid

**DOI:** 10.3390/ijms222312889

**Published:** 2021-11-28

**Authors:** Monika Stompor-Gorący, Maciej Machaczka

**Affiliations:** 1 Department of Human Pathophysiology, Institute of Medical Sciences, University of Rzeszow, Kopisto 2a, 35-959 Rzeszów, Poland; maciej.machaczka@ki.se; 2 Department of Clinical Science and Education, Division of Internal Medicine, Södersjukhuset, Karolinska Institutet, 11883 Stockholm, Sweden

**Keywords:** ferulic acid, nanostructures, encapsulation, antioxidants, neuroprotective action

## Abstract

*Trans*-ferulic acid (FA) is a derivative of 4-hydroxycinnamic acid, which is found in many food products, fruits and beverages. It has scientifically proven antioxidant, anti-inflammatory and antibacterial properties. However, its low ability to permeate through biological barriers (e.g., the blood–brain barrier, BBB), its low bioavailability and its fast elimination from the gastrointestinal tract after oral administration limit its clinical use, e.g., for the treatment of neurodegenerative diseases, such as Alzheimer’s disease. Therefore, new nanotechnological approaches are developed in order to regulate intracellular transport of ferulic acid. The objective of this review is to summarize the last decade’s research on biological properties of ferulic acid and innovative ways of its delivery, supporting pharmacological therapy.

## 1. Introduction

Phenolic compounds are an important group of natural substances of plant origin. The health-promoting properties, such as anticancer, anti-inflammatory, etc., are possessed mainly by antioxidant compounds, and among them are flavonoids and phenolic acids [1,2,3]. For this reason, new methods of their functionalization are developed so as to increase their application in medicine [4,5].

Ferulic acid (FA) is found, among other, in the borage seeds, along with polyphenolic compounds, such as quercetin, galangin and naringenin. Seed extracts of various *Borago* species have high antiproliferative activity to HT-29 human colon cancer cells [6]. It is also an ingredient of wheat bran, where it is found in the form of esters with sugars, such as arabinose [7]. Meanwhile, in the form of glucosides, it is present, among others, in *Nitraria sibirica* [8]. Bioavailability of ferulic acid is dependent on thermal treatment of food products. It was observed that boiling of whole-grain barley varieties enhanced extractability of phenolic acids [9]. Free ferulic acid, including in the form of esters, is also found in the Persian walnut *Juglans regia* [10]. Additionally, it is the main ingredient of foxtail millet phenolic extracts, which are highly active α-glucosidase inhibitors [11]. Moreover, it was identified in the marine brown seaweed *Padina tetrastromatica*, which may be used for development of functional food with antidiabetic and antioxidant activities [12] and in oleoresin from the Curcuma plant, which is a food industry waste [13]. In addition, ferulic acid is found in pectin from sugar beet (*Beta vulgaris*) pulp obtained by extraction with subcritical water [14] and in propolis [15].

**Figure 1:**
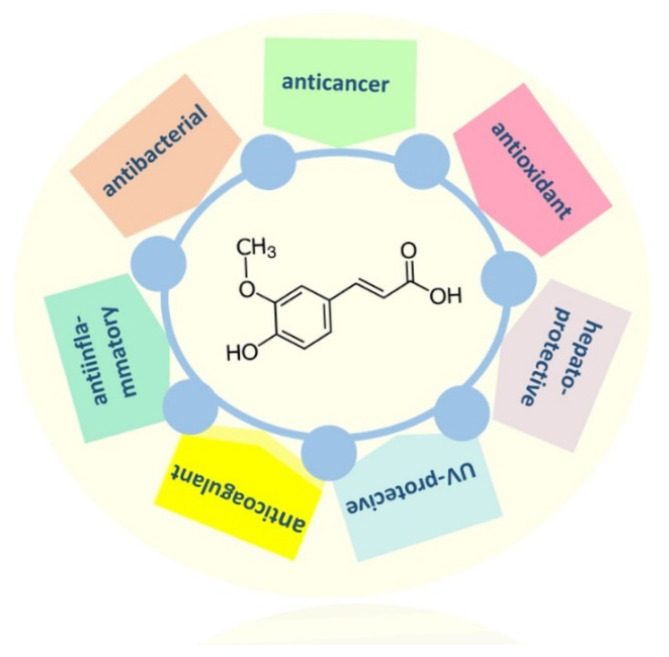
Biological activity of ferulic acid.

From a medical point of view ferulic acid plays an important role in treatment of neurodegenerative diseases, diabetes, cardiovascular diseases, inflammation, and also bacterial and viral infections (Figure 1). It imposes an effect on structures and properties of digestive enzymes, such as pepsin; thus, it may be an important ingredient in formulations of food products for special medical purposes [16].

Ferulic acid and its structural analogues have high antioxidant activity [17]. Being an ingredient of green coffee, it inhibits the activity of monoamine oxidase (MAO)—the enzyme that is responsible for deamination of the central nervous system (CNS) neurotransmitters, such as serotonin (5-HT), for which decreased levels lead to depression and disorders of the satiety center [18]. According to earlier reports, increased levels of serotonin and norepinephrine in the hippocampus and frontal cortex were observed in mice treated with ferulic acid [19]. The compound shows antithrombotic activity [20] and anti-inflammatory properties and has a protective function in eye diseases, such as retinal degeneration (observed after supplementation of mice diet with 50 mg/kg of FA) [21]. It also alleviates acute lung injury through inhibiting the TLR4/NF-κB signaling pathway [22]. Ferulic acid amide derivatives demonstrated in vivo antidiabetic and hypolipidemic effects [23]. In addition, topical application of ferulic acid and its structural analogues may be an efficient and safe method of skin protection against photodamage. FA may be used as an antioxidant to prevent damage from ultraviolet (UV) radiation and skin carcinogenesis [24]. The chemical structure and, resulting from it, physicochemical properties, such as the intercalation into cell membranes, determine the biological activity of ferulic acid [25]. The compound is well soluble in fats and ethanol, whereas its solubility in water is low. Thus, so as to attain the optimal pharmacological activity, new formulations with ferulic acid must be well soluble in water and body fluids. Taking into account the interesting biological properties of ferulic acid and limitations in its medical use, the aim of this review is to sum up the most recent research on pharmacological activity of this compound and new ways of its delivery based on nanocarriers, so as to increase the physiological role of phenolic acids and their derivatives. We summarized the research concerning ferulic acid, its properties and innovative functionalization methods that have been reported over the last decade, in order to facilitate further study on using natural antioxidants, including phenolic acids, in pharmacology and clinical trials. The recent advances in synthesis of some FA prodrugs were also analyzed.

## 2. Main Pharmacological Properties of FA

### 2.1. Detoxification and Hepatoprotective Effects

Naturally occurring methoxylated phenolic acids, including FA, are thoroughly studied with respect to their detoxifying properties, because they may be used for treatment of drug-induced side effects and for preventing toxicity caused for example by polyunsaturated fatty acids, carbon tetrachloride, arsenic or cadmium tetrachloride. Ferulic acid inhibits liver fibrosis progression in non-alcoholic steatohepatitis (NASH) [26]. One of the mechanisms of the activity of FA and its derivatives is also their ability to decrease the levels of proinflammatory cytokines, indicating that FA is beneficial to the immune system [27].

Ferulic acid mitigates arsenic-induced developmental cardiotoxicity [28], ameliorates lead-induced cognitive deficits in vivo [29] and shows protective effect against cadmium chloride–mediated reproductive toxicity [30]. Additionally, it protects the male reproductive system from arsenic-induced toxicity [31] and protects liver cells from the tetrachloride-induced injury [32].

Moreover, ferulic acid has protective effect against cardiac toxicity caused by doxorubicin in rats [33]. Bami et al. [34] demonstrated that treatment with ferulic acid prevents oxidative stress and regulates the levels of BUN (blood urea nitrogen), creatinine, MDA (malondialdehyde), MPO (myeloperoxidase), TOS (total antioxidant status) and PtNT (protein nitrotyrosine) in rats treated with cisplatin. The cardioprotective effect of FA against isoproterenol-induced cardiac toxicity in rats was described by Jain et al. [35].

The studies on the alleviation of arsenic-induced cardiotoxicity have also been documented [28].

The antioxidant activity of ferulic acid leads to the reduction of harmful effects caused by lead acetate, which is an ingredient of many cosmetics, hair dyes and plant protection chemicals [36]. Moreover, the oral administration of ferulic acid to rats fed with a high-fat diet alleviated development of non-alcoholic fatty liver disease (NAFLD) by reducing the deposition of triglycerides and cholesterol in the liver [37]. The compound protects AML-12 hepatocytes against palmitate-induced lipotoxicity by reducing ROS (reactive oxygen species) accumulation and decreasing activation of proinflammatory cytokines, i.e., IL-6 and IL-1β [38]. Therefore, the development of new forms of functional foods enriched with ferulic acid is reasonable in the case of metabolic diseases. The in vivo study with TAC (total antioxidant capacity assay) mice showed that FA exerts a positive effect on gut microbiota, and, in this way, it improves cardiac functions [39]. It also has a modulatory effect on dysregulated redox balance in ferric-induced pancreatic oxidative injury [40].

Ferulic acid can prevent acute liver injury by ameliorating inflammation and regulating GSK-3β/NF-κB/CREB pathway. It also decreases the activity of MPO, aspartate aminotransferase (AST) and alanine aminotransferase (ALT) [41] and alleviates lipopolysaccharide-induced inflammation and acute lung injury in mice [22].

### 2.2. Anticancer Activity

The anticancer activity of natural ferulic acid arises mostly from its capability to suppress reactive oxygen species that protects cellular components, such as DNA, peptides and lipids, from oxidative damage. Moreover, the activity is due to the regulatory effect of FA on intracellular signaling pathways, proliferation, apoptosis and metastasis [42,43,44,45]. In the future, ferulic acid may be an important ingredient of multicomponent formulations, alleviating adverse effects of common chemotherapeutics used in treatment of drug-resistant cancers [46].

It is known that ferulic acid has antitumor activity and that, at high doses, it is less toxic to normal cells than to cancer ones. At a single dose of 300 µg/mL for 10 min, it did not cause any toxicity to platelets (10^3^/mL), leukocytes (10^3^/mL) and erythrocytes (10^6^/mL) from blood samples that were drawn from healthy rodents [20]. There is also evidence that high concentrations of FA (500 and 1000 µM) do not influence the cell viability in 786-O human renal cancer cells [47]. Nevertheless, FA has proven anticancer activity against the cells of human renal adenocarcinoma (ACHN) [48], human urinary bladder carcinoma (T24) [49], human breast cancer (MDA-MB-231) [45] and human osteosarcoma (143B and MG63) [50]. After a 48-h exposure of breast cancer cells (MCF-7) and liver cancer cells (HepG2) to ferulic acid, the measured half-maximal inhibitory concentration values (IC_50_) were 75.4 and 81.38 µg/mL, respectively. Additionally, the observed elevated levels of caspase-8 and -9 indicated induction of apoptosis in the tested cancer cell lines [51]. Moreover, FA at the concentration of 2 mM inhibits proliferation of human cervical cancer cells HeLa and Caski by 88.3 and 85.5%, respectively, inducing cell cycle arrest [52]. Moreover, ferulic acid at a dose of 200 µM inhibits the adhesion and migration of human lung (A549) and colon adenocarcinoma (HT29-D4) cancer cells by 77.9 and 79.8%, respectively [53]. Combination of ferulic acid (10 µM) with drugs, such as epirubicin (1 µM), is a promising therapeutic option for the treatment of breast cancer (MDA-MB-231) [54]. Moreover, the compound enhances the effects of radiotherapy in lung (A549) and liver (HepG2) cancers in later stages of treatment and protects normal lung fibroblasts (WI38) and peripheral blood mononuclear cells (PBMCs) from radiation damage [55]. Moreover, derivatives of FA, due to their strong histone deacetylase inhibitory activity, offer a promising strategy for cancer therapy [56].

### 2.3. Other Properties of Ferulic Acid

Among other significant activities of FA, it is worth noting that it has antimicrobial, photoprotective and anti-inflammatory properties [57,58]. For this reason, the compound is found as an ingredient of dermocosmetic formulations against aging, hyperpigmentation and acne [59]. There are also known neuroprotective [60]) and antidiabetic [61] properties of ferulic acid, including the synergistic interaction of FA with hypoglycemic drugs [62].

Ferulic acid has antimicrobial activity, e.g., towards pathogenic bacteria *E. coli* O157:H7 ATCC 43888 and *L. monocytogenes* ATCC 7644 [63]. For this reason, it finds application as an ingredient of antibacterial packaging materials based on natural polysaccharides. Composite bacterial cellulose–chitosan membranes grafted with ferulic acid may find applications in the food industry as a packaging material to extend the shelf life of food and as dressing materials for slow-healing wounds [64]. Moreover, Liu et al. [65] used ferulic acid for the preparation of innovative composite films used as a packaging material for the preservation of shrimps. Moreover, it was shown that ferulic acid may ameliorate sepsis-induced multi-organ failure. In the in vivo study in rats, the treatment with FA decreased the levels of malondialdehyde (which is an oxidative stress marker), while increasing both the levels of glutathione and the activity of superoxide dismutase and glutathione peroxidase, which protect the organism against the damages of oxidative stress in sepsis [66]. The increased level of oxidative stress may also have an impact on the activity of tyrosinase, the enzyme belonging to the oxidase family. Ferulic acid, in combination with 4-hydroxycinnamic acid, effectively inhibits tyrosinase activity (inhibition rate of 90.44%) [67].

## 3. Novel Strategies for Ferulic Acid Drug Delivery

**Figure 2:**
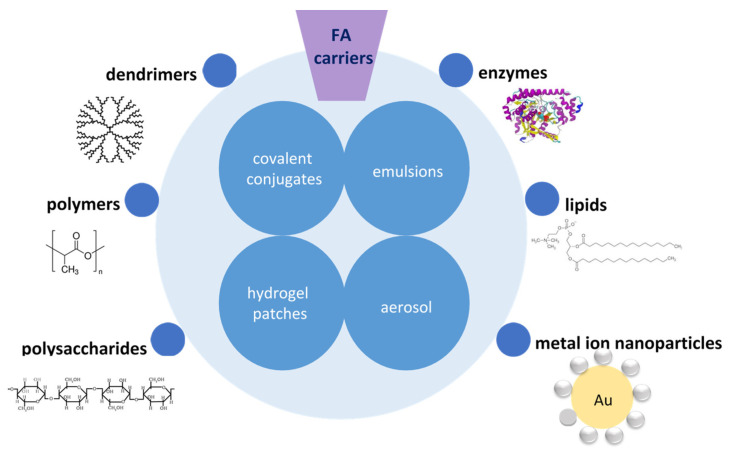
Novel formulations of ferulic acid (FA).

Phenolic antioxidants, such as ferulic acid, have low toxicity and many important physiological functions; therefore, they are widely used in pharmaceutical, food and cosmetic industries. They are free radical scavengers, have affinity for lipid substrates and may be important factors for antioxidant activity. However, their application is limited due to their hydrophobic character and fast decomposition after oral application. For this reason, new ways of its delivery, characterized by sustained release, are being developed (Figure 2). The most common base materials used for the production of novel nanoformulations with FA comprise dendrimers, polymers, certain enzymes, lipids, polysaccharides and also noble metal ions, such as gold.

Qi et al. [68] proposed a novel oxygen delivery system based on hemoglobin modified with ferulic acid that has the capacity to reduce oxidative side reactions and may be used for production of red-blood-cell substitutes.

There is also research on new methods for the functionalization of ferulic acid in order to improve its hydrophilicity. Yao and Sun [69] studied the possibility to use lipases: *Candida antartica* lipase-B, *Candida antartica* lipase-A and *Thermomyces lanuginosus* (Lipozyme TL 100L) for preparation of glyceryl ferulate, which is an easily absorbable form of ferulic acid that is used in cosmetology for the production of dermocosmetics that protect skin from UV irradiation.

An interesting way of simultaneous delivery of antioxidants of different lipophilicity are solid-lipid nanoparticles. Oehlke et al. [70] used the method of hot homogenization to prepare solid-lipid nanoparticles loaded with ferulic acid and tocopherol. The formulations proved to have high antioxidant activity.

One of the innovative methods in modern aesthetic medicine is laser-assisted drug delivery (LAD), which is used to improve the penetration of drugs into the skin, for example, in treatment of scars [71]. A laser-assisted method of delivery of ferulic acid together with vitamins C and E was developed by Waibel et al. [72]. Transdermal delivery of ferulic acid by using microneedle arrays was studied by Yang et al. [73]. The group of Bai et al. [74] prepared transdermal hydrogel patches with ferulic acid on the basis of glycerin, dihydroxyaluminum aminoacetate and tartaric acid. Important for dermatology and cosmetology is the development of innovative triptolide gels with ferulic acid, with potential clinical importance [75]. Meanwhile, aerosol delivery of ferulic acid–loaded nanostructured lipid carriers is a promising approach for the treatment of the respiratory disorders [76].

Del Olmo et al. [77] performed the reaction of amidation-obtained first-generation carbosilane dendrimers functionalized with ferulic acid. These new polyphenolic compounds exhibited higher antioxidant properties than free ferulic acid. The analogous reaction with caffeic and gallic acids gave compounds that inhibited the growth of Gram-positive (+) and Gram-negative (−) bacteria.

Anbazhagan et al. [78] prepared ferulic acid (FA) and paclitaxel (PTX) co-loaded polyamidoamine (PAMAM) dendrimers G 4.5 conjugated with arginyl-glycyl-aspartic acid (RGD) to overcome P-glycoprotein (P-gp)-mediated multidrug resistance (MDR). Ferulic acid delivered in the form of the RGD–PAMAM nanoaggregate enhanced intracellular availability of the medication and induced apoptosis in P-gp-overexpressing multidrug-resistant cells.

A more sophisticated method of increasing FA delivery in topical applications is the development of polymeric nanocarriers. The size of nanoparticles ranges from 10 to 1000 nm, and, most often, they are based on poly(lactic-co-glycolic acid) PLGA. The FA-encapsulated PLGA/PEO nanofibers showed high proapoptotic activity against human breast carcinoma cells (MCF-7) [79].

Rajendran et al. [80] synthesized gold nanoparticles containing ferulic acid as a stabilizing agent (FA-AuNPs), which was next tested for cytotoxicity on human skin cancer cells (A431) and normal keratinocytes (HaCaT). The results confirmed that FA-AuNPs can be used in dermato-oncology in the future, because they induce apoptosis in A431 cells.

Johnson et al. [81] incorporated an FA molecule into fructo-oligosaccharide in order to develop an oral prodrug active against colorectal cancer cells. Additionally, biocompatible hydrogels based on poly-(*N*-isopropylacrylamide) (PNIPAM) and copolymers crosslinked with *N*,*N*-methylenebisacrylamide (BIS) were proposed as scaffold materials for antioxidants [82].

A new way to improve the deficiency of phenolic acids is using a vesicular drug delivery system. Rezaeiroshan et al. [83] designed and prepared niosomes—the vesicles composed of non-ionic surfactants. Recent developments in therapeutic and nutraceutical applications of p-methoxycinnamic acid from plant origin formulations with ferulic acid. Then, the new preparation was evaluated for the in vivo anti-inflammatory activity in rats, using the carrageenan-induced rat paw oedema test. The tested biogel inhibited the oedema by over 20%.

Hassanzadeh et al. [84] designed a nanoformulation based on the silk fibroin as a biomimetic substance coated with neutrophil-membrane-modified ferulic acid. The prepared nanoparticles improved the pharmacological profile of FA and afforded selective delivery of FA into the inflammatory pancreas lesion.

Thermosensitive chitosan/gelatin-based hydrogel containing encapsulated ferulic acid was designed by Wang et al. [85]. The new preparation provided the sustained release of FA. Moreover, it decreased endogenous reactive oxygen species production, improved flow of blood, improved muscle regeneration and decreased inflammation in veins. Therefore, it may be useful in therapy of peripheral arterial disease.

An excellent way to increase the delivery of FA after oral administration was the development of a new nanocarrier system based on chitosan nanoparticles loaded with phospholipid complex (FAPLC CNP) [86]. Another method to improve therapeutic efficacy of ferulic acid may be using aerosolized chitosan nanoparticles, which are supposed to be effective in treatment of asthma [87]. Chitosan–ferulic–bovine serum albumin microcapsules, obtained by the spray-drying technique, demonstrated high thermal stability and in vitro sustained release; thus, they may find application as carriers in novel functional foods, as well as in drug delivery systems [88]. Dermal absorption of ferulic acid delivered in the form of stable w/o/w emulsions with antioxidant properties was developed by the team of Mancuso et al. [89]. The formulation proved capable to treat UV-B-induced erythema.

Amphiphilic polymers of chitosan with ɛ-caprolactone and covalently bonded ferulic acid were prepared for the targeted delivery of antitubercular drugs by Praphakar et al. [90]. Poornima and Korrapati [91] developed innovative nanofibers based on the polycaprolactone-grafted chitosan for the simultaneous delivery of ferulic acid and resveratrol.

Phenolic acids, due to their antioxidant properties, represent an attractive research topic in the field of innovative nutraceuticals. Stable lipid-core nanocapsules based on poly(ε-caprolactone) polymer and loaded with ferulic acid were prepared by Granata et al. [92].

Panwar et al. [93] used an ionic gelation method to prepare chitosan–tripolyphosphate pentasodium (CS–TPP) nanoparticles (NPs) with ferulic acid. The obtained formulation exerted a high antiproliferative effect on human cervical carcinoma cells ME-180.

A promising solution seems to be using a free-radical-induced grafting procedure (ascorbic acid/hydrogen peroxide pair as radical initiator) to prepare chitosan–ferulic acid nanocapsules with application potential [94]. Moreover, ferulic acid in combination with aspirin shows chemopreventive potential towards pancreatic cancer when delivered using chitosan-coated solid-lipid nanoparticles [95].

There is also known a method of preparation of new pharmaceuticals with the use of cellulose acetate as a polymeric matrix for ferulic acid. One of the methods is to prepare a cellulose acetate membrane impregnated with a lipid solution, aiming to mimic skin-barrier function for ferulic acid release [96].

The obtained formulation ensures the sustained release of the active substance [97]. Attempts have also been made to immobilize FA in the solid porous resin Lewatit^®^, which provides the nanoparticles with narrow size distributions [98].

The antioxidant activity of ferulic acid was an inspiration for designing an innovative ophthalmic insert composed of hyaluronan nanofibers and ε-polylysine for the treatment of eye diseases [99]. Similarly, Varela-Garcia et al. [100] designed innovative polymeric contact lenses for the controlled delivery of ocular drugs with ferulic acid. Hydrogels were prepared from the mixtures of 2-hydroxyethyl methacrylate (HEMA), glycidyl methacrylate (GMA) and ethyleneglycolphenylether methacrylate (EGPEM). The proposed solution may be used for the treatment of several eye diseases, including age-related ones. Furthermore, Romeo et al. [101] proposed ferulic acid delivery through polymeric nanoparticles (NPs) consisting of polylactic acid (PLA) and poly(lactic-co-glycolic acid) (NPB) as an effective system for the treatment of eye problems.

A new way of oral FA delivery aiming to reduce the rate of its metabolic conversion and renal elimination, along with increasing its distribution in the brain and improving hypnotic efficacy, was proposed by Liu et al. [102].

In the search for effective ways to control the delivery of antioxidants, whey protein, in combination with maltodextrin, is also studied. Zyaitdinov et al. [103] used this method to encapsulate the polyphenols from oat bran with a high content of ferulic acid. This technique may be used for the preparation of functional food, rich in nutraceuticals. Zein–casein–lysine protein nanoparticles were developed by Reference [104] to modulate the intestinal permeability of ferulic acid and to afford its sustained delivery.

Nanoparticles of ferulic acid and zinc oxide have cytoprotective activity against renal ischemia, which may be due to the enhancement of cell proliferation, upregulation of the antioxidant genes expression (e.g., Nrf2, HIF-1α) or their anti-inflammatory activity (downregulation of TNF-α) [105].

Moreover, in recent years, the development of safe methods of delivery of chemotherapeutics to cancer cells, with the help of non-toxic carriers, has gained more and more importance. Polymers based on natural products may serve as such matrices. Nanoparticles of poly (ferulic acid)-containing doxorubicin (PFA–DOX) demonstrated reduced physical toxicity in vivo compared with free doxorubicin. Additionally, PFA nanocarriers promoted the accumulation of a chemotherapeutic at the tumor site, which supports tumor suppression [106].

Conjugates of ferulic acid with carboxylic curdlans had lower thermal stability and rheological properties than carboxylic curdlans without FA, but their antioxidant activity was very high. Emulsions containing these conjugates showed good β-carotene stability, whereas emulsions without FA did not protect β-carotene from chemical degradation [107].

The o/w emulsions stabilized with ferulic acid–grafted curdlan conjugate (Cur-D-g-FA) were also obtained by Yu et al. [108] in order to improve chemical stability and bioavailability of β-carotene.

Nanocomposite gels based on alginate and prebiotic arabinoxylan materials containing probiotic bacterial cultures *(Lactobacillus plantarum)* and ferulic acid (1.78 µg/g of arabinoxylan oligosaccharides) demonstrated high stability and resistance to gastric conditions [109]. The new nanosystems for ferulic acid delivery are summarized in Table 1.

## 4. Prodrugs of FA

**Figure 3:**
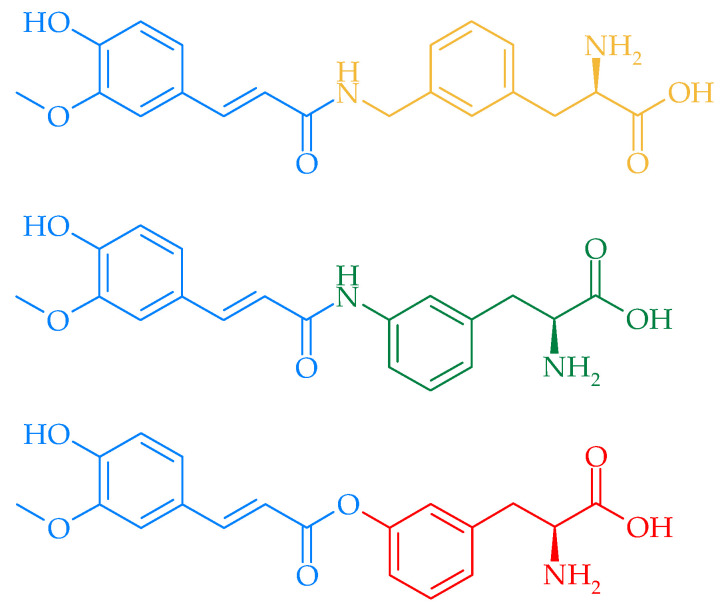
Chemical structures of FA prodrugs based on L-type aminoacids.

Prodrugs are substances that are administered in a pharmacologically inactive form, and after administration, they are metabolized in vivo into the active drugs. When developing prodrugs, researchers focus on the optimization of the absorption, distribution, metabolism and excretion (ADME) properties. An important feature of biologically active substances is their ability to permeate across biological barriers, including getting into the CNS by crossing the blood–brain barrier (BBB). It was observed that amide-based prodrugs of ferulic acid with an aromatic ring were effectively bound to the L-type amino acid transporter (LAT1) (Figure 3) and used the transporter for cellular uptake in vitro and crossed the BBB after in situ perfusion in mice [110]. The amide prodrug with the promoiety directly conjugated in the *meta*-position to ferulic acid underwent the bioconversion to the parent drug in mouse brain. It is worth noting that the analogous ester-based prodrug did bind to LAT1 but did not utilize the transporter for cellular uptake in ARPE-19 cells. However, the presence of an ester linker between the prodrug and the parent drug promoted favorable bioconversion properties in humans.

**Figure 4:**
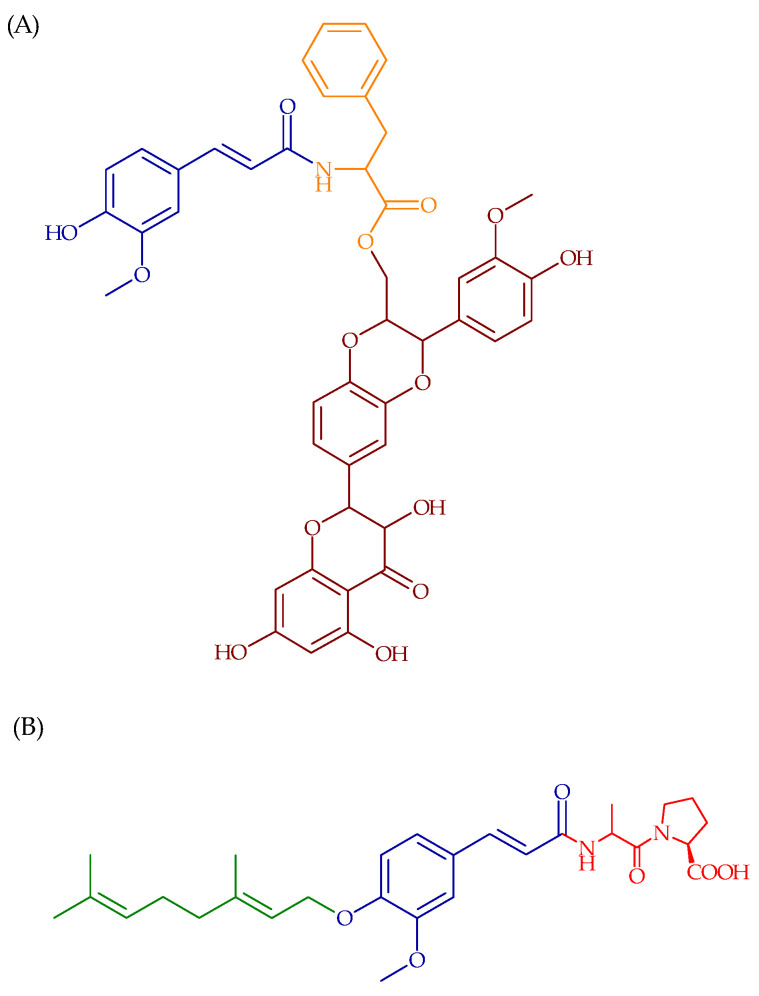
Chemical structures of (**A**) 1-phenylalanine-*N*-(4-hydroxy-3-methoxyphenyl) prop-2-en-*O*-(2R,3R)-3,5,7-trihydroxy-2-((2R,3R)-3-(4-hydroxy-3-methoxyphenyl)-2-(hydroxymethyl)-2,3-dihydro-benzo-(1,4)-dioxin-6-yl)croman-4-one and (**B**) 3-(4′geranyloxy-3′-methoxyphenyl)-2-*trans*-propenoyl-L-alanyl-L-proline.

A new amino acid–based prodrug for simultaneous intestinal release of silybin and ferulic acid was developed by the team of Trombino et al. [111]. The carrier for the synthesized l-phenylalanine-*N*-(4-hydroxy-3-methoxyphenyl) prop-2-en-*O*-(2R,3R)-3,5,7-trihydroxy-2-((2R,3R)-3-(4-hydroxy-3-methoxyphenyl)-2-(hydroxymethyl)-2,3-dihydro-benzo-(1,4)-dioxin-6-yl)croman-4-one was 1-phenylalanine, which has an intrinsic chemical reactivity due to the presence of an amine group, placed on the chiral center, and a carboxylic group. Ferulic acid is attached to the amine group of the amino acid by the amide bond (Figure 4). The obtained prodrug demonstrated high antioxidant activity under simulated physiological conditions; therefore, this method might be used for improving therapeutic potential of other highly reactive and poorly water-soluble biological substances.

Another compound of this group is GAP, a new prodrug of known chemopreventive agent used in the treatment of colon cancer, namely 3-(4′geranyloxy-3′-methoxyphenyl)-2-*trans*-propenic acid (Figure 4), which was investigated by Reference [112]. The results of this study clearly indicate that GAP effectively inhibited colitis-related colon carcinogenesis in mice with no side effects. Dietary GAP had a modulatory effect on cell proliferation by alleviating the oxidative stress (lowering tissue expression and urinary level of 8-OHdG) and enhancing expression of the antioxidant enzyme HO-1. The prodrug of 4′-geranyloxyferulic acid was designed to be hydrolyzed by the intestinal exopeptidase, which specifically hydrolyzes the last peptide bond in tripeptides in which + -Ala (or Gly) and L-pro occupy the second last and last positions, respectively.

Tan et al. [113] designed new PtIV prodrugs of oxoplatin (*cis,cis,cis*-[PtCl_2_(NH_3_)_2_(OH)_2_]), [PtIVCl_2_(NH_3_)_2_(O_2_C-FA)_2_] (Pt-2) and [PtIVCl_2_(NH_3_)_2_(O_2_C-RH)_2_] (Pt-3), by conjugating oxoplatin with ferulic acid and rhein, which have well-known biological activities. Antitumor activity of the new complex compound towards lung cancer cells (A549) and lung cancer xenograft mice model cells (A549/DPP) was higher (67.45% of inhibition) than that of cisplatin (33.05% of inhibition). What is more, the highest concentration of the prodrug was observed in the mitochondria; thus, the proposed anticancer strategy may be a promising approach to personalized anticancer therapy.

There is also research on new phenolipids with amphiphilic properties. Ferulic acid was esterified with butanol to produce butyl ferulate, which was further dihydroxylated, followed by esterification with butyric anhydride to produce a phenolipid containing butyric acid. The obtained phenolipid showed higher antioxidant activity than the substrate (evaluated by the linoleic acid oxidation method) [114].

## 5. Conclusions

Ferulic acid demonstrates multiple pharmacological activities, evaluated both in vitro and in vivo. It has anti-inflammatory and antitumor properties, as well as antidepressant and hepatoprotective ones. Its strong antioxidant activity is used, among others, in the food industry, dermatology and cosmetology. Nevertheless, due to the low lipophilicity, clinical and industrial applications of ferulic acid are limited. Therefore, there is a search for new forms of its delivery in order to increase its bioavailability and a possibility of practical use. The methods of functionalization of ferulic acid include the preparation of prodrugs of FA and development of innovative ways of its delivery. Polysaccharide-based matrices and polymeric nanoparticles are tested as the delivery vehicles. There are developed hydrogels and encapsulates with ferulic acid based on cellulose acetate, hyaluronates, glycerin, dihydroxyaluminum aminoacetate, tartaric acid, lipid nanoparticles and nanoparticles of precious metals, such as gold, which have interesting therapeutic properties.

## Figures and Tables

**Table 1:** The new formulations of ferulic acid.

Formulation	Physicochemical Characteristic	Activity	Reference
FA-SLN Solid-lipid nanoparticles loaded with ferulic acid	Zeta potential: −25 to −43 mV Final FA contents in the SLN: 0.56 and 2.80 mg g^−1^ of dispersion	Stable antioxidant activity	[70]
Laser-assisted method of delivery of ferulic acid together with vitamins C	No data	↑ Wound healing and prevents scarring	[71]
Transdermal hydrogel patches with ferulic acid, on the basis of glycerin, dihydroxyaluminum aminoacetate and tartaric acid	No data	↑ Release of FA from the paste; difficult to permeate through the skin barrier	[74]
FA-loaded NLCs Aerosol delivery of ferulic acid-loaded nanostructured lipid carriers	Particle size: 54.9–148.6 nm Polydispersity index: 0.15–0.37 Zeta potential: (−19.8)–(−25.3) mV Entrapment efficiency: 44.3–94.3%	↑ Period of cytotoxicity time against lung cancer cells (A549); ↑ Pharmacokinetic profile of FA	[76]
Gn-[Si(CH2)3NHC(O)FA]_2_ (G1) Gn-[Si(CH2)3NHC(O)FA]_8_ (G2) First-generation carbosilane dendrimers functionalized with ferulic acid	NMR characterized	No improving the antioxidant activity (DPPH) Antibacterial activity: MIC (ppm) > 16 (*S. aureus* and *E. coli*) % Viability of HFF cells: 95.4 (G1) 92.9 (G2)	[77]
RGD-PAMAM-FP Ferulic acid (FA) and paclitaxel (PTX) co-loaded polyamidoamine (PAMAM) dendrimers G 4.5 conjugated with arginyl-glycyl-aspartic acid (RGD)	Zeta potential: −31.3 mV Size: 144.6 nm	↑ Release of FA; ↑ effectiveness of drug therapy, especially in the treatment of MDR cancers; ↓ P-glycoprotein expression	[78]
FA-encapsulated PLGA/PEO nanofibers	Fiber diameter: 150 ± 47.4 to 200 ± 79 nm	Morphological changes in MCF- 7 cells signs for antiapoptotic effect; ↓ viability of HEK- 293 cells	[79]
FA-AuNPs	Size: 34.2 nm Polydispersity index (PDI) = 0.137	Antiangiogenic properties; encouraged programmed cell death in A431 cells. Proapoptotic: ↓ Mitochondrial membrane potential, Improved the ROS; ↑activation of caspase-3 leading to apoptosis	[80]
Biocompatible hydrogels based on poly-(*N*-isopropylacrylamide) (PNIPAM) and copolymers crosslinked with *N*,*N*-methylenebisacrylamide (BIS)	No data	↑ Antioxidant properties; ↑ time release	[82]
Niosomal biogel of TFA (*trans*-ferulic acid)	EE = 21.64% Particle size: 158.7 nm	Anti-inflammatory effect; inhibited the oedema about 21.37%	[83]
FA-SF-NPs nanoformulation based on the silk fibroin	Size: 186.3 nm PDI: 0.17 Zeta potential: −36.4 mV	↓ Levels of enzymes; prevented the significant enhancement of the inflammatory cytokine levels IL-1β, TNF-α and IL-6; and selective accumulation of FA in the inflammatory lesions of the pancreas	[84]
FA-gel chitosan/gelatin-based hydrogel containing encapsulated ferulic acid	Gelation time: 64.75 ± 3.31 s at 37 °C	Antioxidant effect; decreasing endogenous reactive oxygen species production, inflammation-related gene expression and apoptosis level; improves blood flow and muscle regeneration; and decreases inflammation in veins	[85]
Chitosan nanoparticles loaded with phospholipid complex (FA-FAPLC CNP)	Particle size ~123.27 nm, PDI value ~0.31 Zeta potential: ~32 mV Spherical-shaped morphology	↑ Aqueous solubility of FA around ~(12-fold), ↑ antioxidant activity and ↑ oral bioavailability	[86]
Aerosolized hyaluronic acid decorated, ferulic acid–loaded chitosan nanoparticles	Size: 164.2 ± 9.7 nm Zeta potential: (24.0 ± 0.5 mV) Entrapment efficiency: (EE%) (65.0 ± 1.5) Loading capacity: (LC%) (18.5 ± 0.4) Mass median aerodynamic diameter (MMAD) of 1.81 ± 0.15 µm	↑ Interaction and transportation across mucus barrier	[87]
Ferulic acid delivered in the form of stable w/o/w emulsions		↑ Percutaneous permeation; possible topical application in photo-induced erythema	[89]
CS-g-PCL/FA chitosan with ɛ-caprolactone and covalently bonded FA	Average size: 100–210 nm	Potential for delivery of hydrophobic antitubercular drugs	[90]
FA-chitosan-polycaprolactone nanofibers	Size: 200–240 nm	Antioxidant activity Cytocompatible and able to provide sustained Release of bioactive to support keratinocytes growth in vitro non-hemolytic activity Improve keratinocytes migration in vitro	[91]
FA-NC nanocapsules based on poly(ε caprolactone) polymer, loaded with FA	Nanoparticles loaded with hydroxycinnamic acids (HA-NCs) have diameter of 224–253 nm, encapsulation efficiency of 53–78%, and are stable over time (30 days). Zeta potential: −7 mV EE: 62% pH: 4.2 PDI: 0.08 FA loaded amount: 0.62 mg/mL	Protect the HAs in simulated gastric fluid (SGF) and release them in simulated intestinal fluid (SIF)	[92]
FA/CS–TPP NPs chitosan–tripolyphosphate pentasodium (CS–TPP) nanoparticles (NPs) with ferulic acid	No data	Antiproliferative activity against ME-180 cells	[93]
Microencapsulates of BSA with ferulic acid–grafted chitosan	Primary absorption peak at 350 nm	↑ Sustained-release effection	[94]
Chitosan-coated solid-lipid nanoparticles	Particle sizes: 183 ± 46 and 229 ± 67 nm Encapsulation efficiency of 80 and 78% Zeta potential of 39.1 and 50.3 mV	Chemopreventive effects on 40-fold decreases in dose of FA against human pancreatic cancer cells MIA PaCa-2 and Panc-1 suppressed the growth of the tumor by 45%; decrease expression of proliferation proteins PCNA and MKI67; and also increased expression of apoptotic proteins p-RB, p21 and p-ERK1/2	[95]
FA-cellulose acetate nanostructures	Average diameter of 760 ± 130 nm	Drug loading: 71.5%	[97]
FA-Lewatit^®^ Immobilize FA in the solid	Changes in the FTIR-ATR peaks 1685/cm (FA) 1267/cm (C=O) and 1184/cm (O–C)	Average release of 32 mg FA/g of dry loaded resin (recovery of 22%)	[98]
FA-NA-ε-PL-PVP Hyaluronan nanofibers and ε-polylysine	mean thickness of 270 ± 21 µm and 273 ± 41 µm	Innovative ophthalmic insert composed of hyaluronan (HA) nanofibers for the dual delivery of an antioxidant (ferulic acid, FA) and an antimicrobial peptide (ε-polylysine, ε-PL) antibacterial activity: *Pseudomonas aeruginosa* and *Staphylococcus aureus*	[99]
FA-loaded G400E200-0 and G400E200-C hydrogels Hydrogels functionalized with the nitrogenous base cytosine for the controlled uptake and release of transferulic acid (TA)	FRIR strong band at 1655 cm^−1^ (amide carbonyl group)	↑ Accumulation of FA in cornea and sclera tissues	[100]
Polymeric nanoparticles (NPs) consisting of polylactic acid (NPA) and poly(lactic-co-glycolic acid) (NPB)	FA-NPAs: Size: 178 nm PDI: 0.056 Zeta potential: −33.7 mV FA-NPBs: Size: 219 nm PDI: 0.207 Zeta potential: −23.80	Promising carriers for ocular drug delivery	[101]
Self-microemulsifying drug delivery system: FA-loaded SMEDDS	Droplet size: 15.24 nm	Oral bioavailability: 185.96% Higher distribution in the brain and enhanced serotonin levels in the brain Extended the sleep time by 2-fold and has good stability	[102]
Zein-casein-lysine protein-FA-nanoparticles	Size: 199 nm Zeta potential: −26 mV	Modulate the intestinal permeability of FA Prolonged FA release safe profile against Caco-2 and HT29-MTX cells	[104]
Combination FA and ZnO-NPs	No data	Significant improvement in the elevated serum creatinine and BUN and MDA concentrations and expression of TNF-α, Bax and caspase-3 in kidney tissues Rise in the creatinine clearance, the activities of catalase (CAT) and superoxide dismutase (SOD) and the expression of HO-1, HIF-1α genes and proliferation marker (ki67) in kidney tissues	[105]
PFA–DOX NPs nanoparticles of poly(ferulic acid) containing doxorubicin	No data	Accumulation and retention at the tumor site Superior tumor suppression. Improving safety Reduced the physical toxicity of free DOX	[106]
FA-grafted curdlan conjugate (Cur-D-g-FA)	Zeta potential: −22.57–(−34.87) mV	Favorable bioaccessibility of BC in vitro oxidation stability	[108]

↓/↑—decrease/increase in activity.

## Data Availability

Data are contained within the article.
